# Conventional and Novel Inflammatory Biomarkers in Chronic Heart Failure Patients with Atrial Fibrillation

**DOI:** 10.3390/medicina60081238

**Published:** 2024-07-30

**Authors:** Gregor Vercek, Borut Jug, Marko Novakovic, Miha Antonic, Anze Djordjevic, Jus Ksela

**Affiliations:** 1 Department of Vascular Diseases, University Medical Centre Ljubljana, 1000 Ljubljana, Slovenia; gregor.vercek@kclj.si (G.V.); borut.jug@kclj.si (B.J.); marko.novakovic@kclj.si (M.N.); 2 Faculty of Medicine, University of Ljubljana, 1000 Ljubljana, Slovenia; 3 Department of Cardiac Surgery, University Medical Centre Maribor, 2000 Maribor, Slovenia; miha.antonic@ukc-mb.si (M.A.); anze.djordjevic@ukc-mb.si (A.D.); 4 Faculty of Medicine, University of Maribor, 2000 Maribor, Slovenia; 5 Department of Cardiovascular Surgery, University Medical Centre Ljubljana, 1000 Ljubljana, Slovenia

**Keywords:** atrial fibrillation, heart failure, interleukin-6, interleukin-6/interleukin-10 ratio, orosomucoid, endocan

## Abstract

(1) *Background and Objectives:* Atrial fibrillation (AF) is the most common cardiac arrhythmia and is associated with increased morbidity and mortality both in the general population and heart failure patients. Inflammation may promote the initiation, maintenance and perpetuation of AF, but the impact of inflammatory molecular signaling on the association between AF and heart failure remains elusive. (2) *Materials and Methods:* In 111 patients with chronic stable heart failure, baseline values of conventional (IL-6 and hsCRP) and selected novel inflammatory biomarkers (IL-10, IL-6/IL-10 ratio, orosomucoid and endocan) were determined. Inflammatory biomarkers were compared with respect to the presenting cardiac rhythm. (3) *Results:* Patients aged below 75 years with AF had significantly higher values of IL-6 and IL-6/IL-10 ratio; IL-6 levels were a significant predictor of AF in both univariate (OR 1.175; 95%CI 1.013–1.363; *p* = 0.034) and multivariate logistic regression analysis when accounting for other inflammatory biomarkers (OR 1.327; 95% CI 1.068–1.650; *p* = 0.011). Conversely, there was no association between other novel inflammatory biomarkers and AF. (4) *Conclusions:* IL-6 levels and the IL-6/IL-10 ratio are associated with AF in patients with chronic stable heart failure under the age of 75 years, suggesting that inflammatory molecular signaling may play a role in the development of AF in the heart failure population.

## 1. Introduction

Atrial fibrillation (AF) is the most common sustained cardiac arrhythmia with gradually increasing incidence and prevalence globally. It is strongly associated with increased morbidity and mortality, consequently imposing a significant burden on healthcare resources worldwide [1].

A growing body of evidence has accumulated over the last few decades, indicating that AF is not only strongly related to a variety of molecular derangements (e.g., Ca^2+^-dependent intra-cellular processes, microRNA changes and Na+ channel dysfunction) but also to inflammatory signaling [2,3,4,5]. The latter mainly involves potent inflammatory signaling molecules, such as interleukin (IL)-6 [6,7,8,9,10,11] and high-sensitivity C-reactive protein (hsCRP) [2,3,12]. In particular, IL-6 has been suggested as a promotor of AF development through electrophysiological and structural changes in cardiomyocytes, which are brought about by the IL-6 action of NLRP3 inflammasomes and connexin channel-dependent pathways [13,14,15,16]. Furthermore, several novel inflammatory markers have lately been associated with the incidence and progression of various cardiovascular pathologies, including heart rhythm disturbances, such as anti-inflammatory IL-10, orosomucoid (i.e., alpha-1-acid glycoprotein, an acute phase protein) and endocan (a soluble chondroitin/dermatan sulfate proteoglycan, expressed in response to inflammatory cytokines and affecting the expression of cell adhesion molecules) [17,18,19,20,21]. 

Today we know that AF is strongly associated with heart failure (HF)—both conditions often coexist and worsen each other, with AF conferring an increased risk of all-cause mortality across all HF subtypes [22]. In addition to cardiovascular risk factors, underlying structural diseases and functional cardiac abnormalities, several other determinants may contribute to AF in HF populations, including HF-associated inflammation. Although distinctive molecular pathways related to the abovementioned inflammatory signaling molecules have been shown to play a significant role in AF onset and progression in non-HF populations, their study in HF patients with AF has been limited [17,23,24]. 

Thus, the purpose of this study was to evaluate the relationship between both established and novel inflammatory biomarkers and AF in patients with stable chronic HF.

## 2. Materials and Methods

### 2.1. Patients, Study Design and Biochemical Analysis

**Figure 1:**
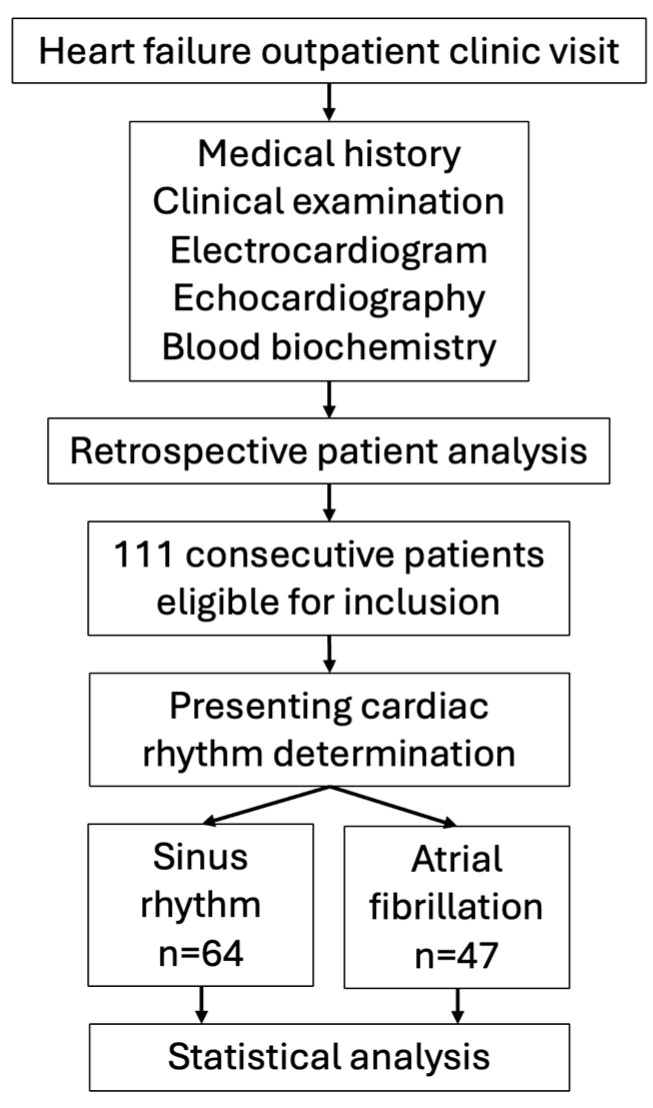
Study outline.

Consecutive patients with chronic stable HF were recruited from the heart failure outpatient clinic of the Department of vascular diseases of the University Medical Centre Ljubljana, Slovenia (Figure 1). The methodology of the study was already previously reported [25,26]. In summary, patients were included if they presented with clinical and echocardiographic evidence of left ventricular dysfunction (either with a left ventricular ejection fraction (EF) < 50% according to the Simpson biplane method, or left ventricular EF > 50% plus a mitral ring E/E′ ratio > 15 or an E/E′ ratio > 8 with the addition of one of the following: atrial fibrillation, elevated natriuretic peptides or echocardiographic parameters of diastolic dysfunction on transmitral and pulmonary venous flow patterns) and were classified in stages II and III according to the New York Heart Association (NYHA) classification. Only patients with stable chronic HF were included without recent acute cardiovascular events, such as acute myocardial infarction, stroke or thromboembolic events, in the previous 3 months prior to inclusion. Additionally, patients with significant liver (liver transaminase levels > 3× upper normal limit) or renal dysfunction (serum creatinine > 250 mcg/L), acute or chronic inflammatory diseases or malignancy were excluded. 

At inclusion, a thorough clinical examination, electrocardiogram (ECG) recording, echocardiographic assessment and blood biochemistry analysis were performed in all patients. For this study, the patient cohort was divided in two groups according to the presenting ECG rhythm—either sinus rhythm or AF (including paroxysmal, persistent and permanent AF). The two groups were compared with respect to patient characteristics (age, sex, comorbidities and medications), laboratory biomarkers (N-terminal pro-brain natriuretic peptide (NT-proBNP), hsCRP, IL-6, IL-10, IL-6/IL-10 ratio, orosomucoid, endocan) and left ventricular EF. 

Venous blood was sampled from the cubital vein in the supine position after 30 min of rest. The collected blood samples were centrifuged (for 10 min at 3000 RPM and 0 °C) and immediately separated. The analytical methods for the measurement of inflammatory biomarkers were laser nephelometric technique for hsCRP (Behring Diagnostics, Rarburg, Germany), enzyme-linked immunosorbent assay (ELISA) for IL-6, IL-10 and orosomucoid (R&D Systems, Minneapolis, MN, USA) and ELISA for endocan measurement (Lunginnov^®^ Systems, Lille, France). The study was conducted with the approval of the Ethics Committee of the Republic of Slovenia (No. 101/02/14) and in compliance with the Declaration of Helsinki. Written informed consent was obtained from all patients prior to inclusion in the study.

### 2.2. Statistical Analysis

The normality of the distribution of continuous variables was assessed with the Shapiro–Wilk test. Baseline characteristics are presented as means (standard deviation) for normally distributed continuous variables, as medians (interquartile range, IQR) for non-normally distributed continuous variables and by frequency (percentage) for categorical variables. Between-group differences were assessed with the Mann–Whitney U test for continuous variables because of deviation from the normality of most continuous variables, and with the Chi-square test for categorical variables. The correlation between presenting rhythm and predictors was assessed with univariate and multivariate logistic regression analysis. Logistic regression analysis results are presented as odds ratios (ORs) with the corresponding 95% confidence intervals (CIs). Subgroup analysis was performed by a further division of the patient cohort according to age below or over 75 years. An age cut-off of 75 years was chosen for subgroup analysis to assess the relationship between inflammatory markers and AF before other age-dependent factors might prevail, since epidemiological studies have indicated that AF incidence peaks between 75 and 79 years of age [27]. A two-tailed *p*-value equal to or below 0.05 was considered as statistically significant. Statistical analysis was performed using JASP version 0.18.1 (JASP team, Amsterdam, The Netherlands).

## 3. Results

The main results can be summarized as follows:

- AF is associated with elevated levels of IL-6 and a higher pro-/anti-inflammatory IL-6/IL-10 ratio in patients with stable chronic HF under the age of 75 years.
- IL-6 levels were independently associated with AF even after adjusting for other inflammatory biomarkers.
- There was no association between the novel inflammatory markers orosomucoid or endocan and AF in patients with chronic stable HF.

A total of 111 patients with chronic stable HF were included in the study and 42.3% had AF. The majority of patients were male (64.9%). Mean age was 71.2 ± 10.7 years and the median EF was 35% (IQR 27.5–40%). Patients with AF and sinus rhythm were well balanced in most baseline characteristics, including NYHA class, comorbidities and secondary preventive and heart failure medication (Table 1). The only exceptions were the use of loop diuretics and digoxin, which were more common in patients with AF, and lipid-lowering therapy with statins, which was more common in patients with sinus rhythm. The study was conducted before sodium-glucose co-transporter-2 (SGLT-2) inhibitors were approved for heart failure patients. With respect to blood biochemistry, patients with AF had significantly higher values of NT-proBNP (2095 vs. 1352 pg/mL, *p* = 0.046); the observed differences in other parameters were not significant.

**Figure 2:**
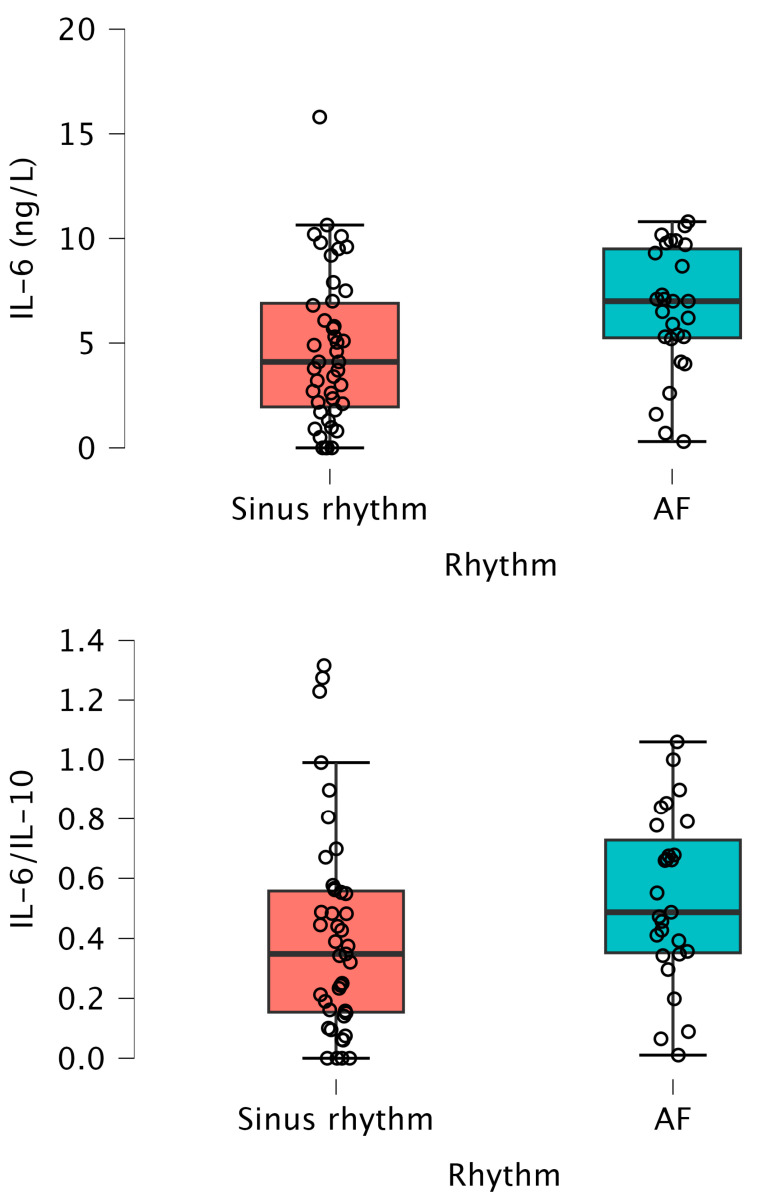
(**Top**) Box plot for IL-6 with regard to presenting rhythm in patients ≤ 75 years, *p* = 0.012. (**Bottom**) Box plot for IL-6/IL-10 ratio with regard to presenting rhythm in patients ≤ 75 years, *p* = 0.044. Abbreviations: AF—atrial fibrillation and IL—interleukin.

Regarding inflammatory biomarkers, there was no significant association between hsCRP, IL-6, IL-10, IL-6/IL-10 ratio, orosomucoid or endocan levels and the presenting cardiac rhythm (Table 1). When further subgroup analysis was performed with respect to age ≤75 years (43 patients in sinus rhythm and 27 patients with AF), we observed statistically significantly higher values of NT-proBNP, IL-6 and IL-6/IL-10 ratio in patients with AF in comparison to patients in sinus rhythm (Table 2, Figure 2). 

In logistic regression analysis, there was a significant positive correlation only between IL-6 and AF in both univariate logistic regression (OR 1.175; 95% CI 1.013–1.363; *p* = 0.034) and multivariate logistic regression analysis when accounting for NT-proBNP, hsCRP, IL-10, orosomucoid and endocan (OR 1.327; 95% CI 1.068–1.650; *p* = 0.011). There was no significant correlation between other abovementioned inflammatory biomarkers and AF in either univariate or multivariate logistic regression analysis (Table 3). Conversely, in patients over 75 years of age, there were no significant differences in laboratory biomarkers when comparing the two groups and no correlation between AF and laboratory biomarkers in regression analysis.

Since the rates of statin and digoxin therapy were different in the sinus rhythm and AF patient cohorts (Table 1), we performed additional analyses for the assessment of any potential association between statin or digoxin therapy and inflammatory biomarkers. There were no significant differences in hsCRP, IL-6, IL-10, orosomucoid or endocan levels with respect to statin therapy in both overall and ≤75 years patient cohorts. Conversely, digoxin therapy was associated with significantly higher IL-6 levels both overall (6.8 (4.7–8.9) ng/L vs. 4.1 (2.1–7.4) ng/L, *p* = 0.033) and ≤75 years of age (6.8 (5.7–9.4) ng/L vs. 4.8 (2.0–7.4) ng/L, *p* = 0.031), and with higher levels of endocan in the overall cohort (3.8 (3.1–6.0) mcg/L vs. 3.2 (2.2–4.6) mcg/L, *p* = 0.036), but not ≤75 years. There were no significant differences in other inflammatory markers overall or in patients aged ≤75 years with respect to digoxin therapy.

## 4. Discussion

Our pilot study of the relationship between inflammatory biomarkers and AF has shown that AF is associated with increased levels of IL-6 and a higher pro-/anti-inflammatory IL-6/IL-10 ratio in patients with stable chronic HF under the age of 75 years. IL-6 levels were independently associated with AF even after adjusting for other inflammatory biomarkers. Conversely, other selected inflammatory biomarkers—namely hsCRP, IL-10, orosomucoid and endocan—were not significantly different between patients in AF and patients in sinus rhythm.

Our study adds to the growing body of evidence on the association between AF and inflammation [2,3,4]. Chronic inflammation represents the intersection between HF and AF, and is involved in both the initiation and maintenance of AF through electrical and structural changes in the atria [28,29]. Most previous studies focused on IL-6 and demonstrated that IL-6 is associated with AF in post-menopausal women [30], patients with stable coronary artery disease [7], in individuals following coronary artery bypass surgery [8], and in patients with chronic kidney disease [9]. Furthermore, IL-6 is a predictor of AF recurrence [10,11], and AF associated long-term adverse events [31,32]. Several molecular mechanisms have been identified in the pathophysiology of AF [5]. One of these pathways is centered around the NLRP3 inflammasome [5,13,14]. It is part of a central innate immunity signaling pathway spanning from the NLRP3 inflammasome through IL-1 to IL-6 [33]. Increased NLRP3 activity was observed in patients with both paroxysmal and chronic AF, and leads to electrophysiologic abnormalities, atrial structural changes and abnormal calcium release from the sarcoplasmic reticulum in animal models [13,14]. One of the newly identified mechanisms linking IL-6 with AF development is concerted by connexins, gap junction proteins, which are expressed in the atria and affect their electrophysiological properties, thereby influencing AF maintenance [15]. Elevated IL-6 levels trigger a reduction of circulating connexin levels, which in turn alters the connexin channel-dependent pathways, leading to rapid atrial electrical remodeling [16]. Specifically, increased P-wave dispersion indices were noted in patients with active inflammation [16]. Additionally, there was an inverse relationship between both P-wave dispersion indices and IL-6 on one hand, and circulating connexin levels on the other hand, which in turn reflected atrial connexin expression [16]. The association between IL-6 and reduced connexin expression was later confirmed in a cellular model of HL-1 mouse atrial myocytes [16]. Conversely, IL-10 is an anti-inflammatory cytokine and was shown to reduce atrial remodeling and fibrosis in mice [34]. Thus, the IL-6/IL-10 ratio represents a balance be-tween pro- and anti-inflammatory cytokines and may reflect structural changes in the atria. In individuals undergoing cardiopulmonary bypass, a higher IL-6/IL-10 ratio was associated with a higher incidence of postoperative AF in comparison to patients with an attenuated IL-6/IL-10 response due to hydrocortisone administration [19]. In this regard, our findings are in line with previous reports showing that inflammation may also play a role in the pathophysiology of AF in patients with HF. However, the independent association between IL-6 and AF in our study was limited to patients below the age of 75 years. On the one hand, advanced age may overwhelm any possible impact of inflammatory signaling on the association between AF and HF. On the other hand, in younger individuals with HF, inflammation-related mechanisms may represent an additional determinant for the initiation, maintenance and perpetuation of AF. 

A recently published study of 105 patients with non-valvular AF evaluated a variety of different inflammatory markers, both established and novel [35]. Only IL-6, IL-10, tumor necrosis factor (TNF) and interferon-gamma-induced protein 10 (IP-10) showed an association with AF after multivariate regression analysis; however, the study did not report a separate analysis for patients with HF [35]. Conversely, a study of 78 patients with stable HF reported on the association of elevated IL-6 levels with a higher prevalence of AF, as well as with higher mortality and hospital readmissions [36]. The study mostly included patients with preserved EF. This is in contrast with our study, where most patients had HF with reduced EF. Additionally, we aimed to assess the relationship between both established as well as novel inflammatory biomarkers and AF in the HF population. However, novel inflammatory biomarkers, namely hsCRP, orosomucoid and endocan, ultimately did not emerge as predictors of AF in our study. In terms of hsCRP, studies have shown that IL-6 is a superior marker of inflammation to hsCRP [25], which was also the case in our analysis. hsCRP is generally elevated in HF patients and may be lacking the requisite sensitivity to detect significant differences between patients with AF and sinus rhythm in the HF patient population [37]. Similarly, several studies have indicated that hsCRP in HF patients can robustly predict the overall mortality [38], but might lack the ability to predict more subtle clinical changes, especially in individuals with HF with reduced EF [39], which were mostly included in this study. In terms of orosomucoid, previous studies have shown that elevated levels may be associated with incident AF after cardiac surgery in women [23], but were not associated with the risk of AF-related hospitalization in the general population [24]. On a tissue level orosomucoid is released not only from the liver, but also from epicardial adipose tissue, which has been shown to be a contributing factor in the initiation, maintenance and perpetuation of AF [20,40]. Orosomucoid has been shown to influence adipose tissue extracellular matrix remodeling by inhibiting adipose tissue fibrosis through AMP-activated protein kinase activation [41]. In terms of endocan, previous studies suggest that elevated levels may be associated with AF in asymptomatic individuals [17]. Endocan is an inflammatory mediator produced by the endothelium and increases the expression of soluble intercellular adhesion molecules 1 (ICAM-1) and vascular cell adhesion molecule-1 (VCAM-1), both of which have been associated with AF [21,42,43,44]. Although previous evidence suggests that orosomucoid and endocan-driven pathways may be involved in AF onset and progression in non-HF patient populations [17,23], our study found no association between levels of novel inflammatory signaling molecules and AF in patients with chronic stable HF. Our results indicate that selected novel biomarkers may not possess the ability to differentiate between patients with AF and patients in sinus rhythm in the HF population and are thus in this regard inferior to well established inflammatory markers, such as IL-6. However, whether selected AF-related inflammatory signaling pathways are importantly altered in HF patients or only masked by other distinctive HF-related molecular derangements remains elusive and further studies are warranted to provide more definite answers. 

On the other hand, HF is itself associated with inflammation [45]. It is characterized by elevated pro-inflammatory cytokines, as well as with the activation of components of innate and adaptive immunity [46,47]. Additionally, different HF phenotypes are associated with distinct biomarker profiles, with inflammation playing a more prominent role in HF with preserved EF [48]. IL-6, which was, in our study, associated with AF in patients aged ≤75 years, is elevated in patients with HF, and also correlates with disease severity and prognosis [46]. Higher levels of IL-6 in younger patients with AF in our study may therefore in part reflect a higher degree of inflammation in the context of more advanced heart failure. In the present study patients with AF and sinus rhythm also differed in the rates of statin and digoxin therapy. Statin therapy was more common in patients with sinus rhythm, whereas the rate of digoxin therapy was higher in patients with AF. Both statins and digoxin are known to have immunomodulatory effects and reduce the concentration of IL-6 [49,50]. In comparison, in our study statins did not have an impact on inflammatory markers, while digoxin was paradoxically associated with increased IL-6 levels, probably reflecting higher AF burden, since digoxin therapy was more common in the AF patient cohort.

Our study provides evidence on the association between established inflammatory biomarkers and AF in HF patients aged under 75 years. In this respect, inflammation can be regarded as a marker of AF risk in younger patients with HF and thus a possible target for a better risk-stratification of HF patients. However, several limitations require caution in interpreting our results. Firstly, this was a cross-sectional study to find association, whereas a prospective cohort study in the future may provide a less biased appreciation of the clinical risk of AF occurrence in relation to inflammatory biomarkers in patients with HF. Secondly, due to the exploratory nature of the study, a relatively small sample size and single-center design it is impossible to generalize the results to the entire HF population. Additionally, the study may have been underpowered to detect significant associations between other inflammatory biomarkers and AF. The small sample size also prevented us from performing further subgroup analysis with respect to sex, AF type (paroxysmal, persistent or permanent) and HF phenotype (HF with preserved, mildly-reduced or reduced EF), which might potentially influence the results of our study. Thirdly, while patients were enrolled in the study prospectively, the present analysis was done retrospectively, and some patients were excluded due to incomplete data. Finally, patients with AF had higher values of NT-proBNP and therefore more advanced heart failure, which could account for greater inflammation. Even though we demonstrated a correlation between IL-6 and AF, our findings do not provide the exact pathophysiological mechanism of the complex interplay between HF, inflammation and AF.

## 5. Conclusions

In conclusion, we have shown that IL-6 levels and the IL-6/IL-10 ratio are associated with AF in patients with stable chronic HF under the age of 75 years. Our findings suggest that inflammation may play a contributing role in the development of AF in HF patients below the age of 75 years, whereas age-related factors may overpower and/or mask the impact of inflammatory molecular signaling in older HF individuals. Additionally, our results indicate that selected novel biomarkers may not possess the ability to differentiate between patients with AF or sinus rhythm in the HF population and are thus in this regard inferior to well established inflammatory markers. Larger studies are needed to further elucidate the role of inflammation in the development of AF in the HF population.

## Figures and Tables

**Table 1:** Baseline patient characteristics.

	All	Sinus Rhythm	AF	*p*-Value
Patients (n)	111	64 (57.7%)	47 (42.3%)	/
Age (years)	71.2 ± 10.7	69.6 ± 11.4	73.5 ± 9.4	0.116
Sex (male, %)	64.9	68.8	59.6	0.317
AF (%)	42.3	0	100	/
EF (%)	35 (27.5–40)	34 (27–40)	35 (30–40)	0.206
CAD (%)	49.6	54.7	42.6	0.206
CVD (%)	22.5	20.3	25.5	0.515
PAD (%)	19.8	18.8	21.3	0.741
DM (%)	33.3	34.4	31.9	0.786
AH (%)	71.2	73.4	68.1	0.538
HLP (%)	47.7	53.1	40.4	0.186
NYHA I/II/III (%)	3.6/65.8/30.6	6.3/68.8/25.0	0/61.7/38.3	0.095
ACEI/ARB (%)	97.3	98.4	95.7	0.387
Beta blocker (%)	90.1	87.5	93.6	0.287
MRA (%)	50.5	45.3	57.4	0.206
Loop diuretic (%)	64.0	56.3	74.5	0.048
Digoxin (%)	21.6	9.4	38.3	<0.001
Statin (%)	37.8	46.9	25.5	0.022
NT-proBNP (pg/mL)	1697 (705–4353)	1352 (494–4670)	2095 (1423–3992)	0.046
hsCRP (mg/L)	2.7 (1.4–6.6)	2.9 (1.4–6.2)	2.4 (1.4–7.5)	0.988
IL-6 (ng/L)	5.1 (2.5–8.2)	3.9 (2.1–7.6)	6.1 (3.5–8.9)	0.083
IL-10 (ng/L)	11.6 (9.4–13.7)	11.2 (9.0–13.5)	12.1 (9.9–14.2)	0.233
IL6-/IL-10	0.428 (0.187–0.674)	0.382 (0.158–0.645)	0.488 (0.320–0.713)	0.160
Orosomucoid (mcg/L)	597 (392–1062)	602 (434–952)	566 (349–1223)	0.818
Endocan (mcg/L)	3.3 (2.4–4.9)	3.2 (2.3–4.6)	3.6 (2.9–5.0)	0.300

ACEI—angiotensin converting enzyme inhibitor, AF—atrial fibrillation, AH—arterial hypertension, ARB—angiotensin receptor blocker, CAD—coronary artery disease, CVD—cerebrovascular disease, DM—diabetes mellitus, EF—ejection fraction, HLP—hyperlipidemia, hsCRP—high-sensitivity C-reactive protein, IL—interleukin, MRA—mineralocorticoid receptor antagonist, NYHA—New York Heart Association class, NT-proBNP—N-terminal pro-brain natriuretic peptide and PAD—peripheral artery disease.

**Table 2:** Biomarker values in patients ≤ 75 years.

	All	Sinus Rhythm	AF	*p*-Value
Patients (n)	70	43	27	
NT-proBNP (pg/mL)	1563 (570–4583)	729 (332–4447)	2095 (1491–4764)	0.022
hsCRP (mg/L)	2.8 (1.3–6.7)	2.8 (1.5–6.5)	2.4 (1.2–7.9)	0.914
IL-6 (ng/L)	5.3 (2.6–7.8)	4.1 (2.0–6.9)	7.0 (5.3–9.5)	0.012
IL-10 (ng/L)	12.2 (9.5–14.3)	12.0 (9.3–13.7)	12.4 (10.8–16.6)	0.178
IL-6/IL-10	0.427 (0.192–0. 663)	0.348 (0.154–0.558)	0.488 (0.352–0.728)	0.044
Orosomucoid (mcg/L)	604 (371–1033)	550 (383–959)	649 (322–1431)	0.638
Endocan (mcg/L)	3.2 (2.2–4.4)	2.9 (2.2–4.0)	3.4 (2.3–4.8)	0.242

AF—atrial fibrillation, hsCRP—high-sensitivity C-reactive protein, IL—interleukin and NT-proBNP—N-terminal pro-brain natriuretic peptide.

**Table 3:** Logistic regression analysis results in patients ≤ 75 years.

	Univariate Logistic Regression		Multivariate Logistic Regression	
	OR (95% CI)	*p*-Value	OR (95% CI)	*p*-Value
IL-6	1.175 (1.013–1.363)	0.034	1.327 (1.068–1.650)	0.011
IL-10	1.002 (0.959–1.047)	0.931	0.998 (0.953–1.044)	0.914
IL-6/IL-10	3.435 (0.745–15.836)	0.113	NA	NA
Orosomucoid	1.000 (1.000–1.001)	0.319	1.000 (0.999–1.001)	0.645
Endocan	1.151 (0.875–1.515)	0.315	1.039 (0.767–1.407)	0.805
NT-proBNP	1.000 (1.000–1.000)	0.782	1.000 (1.000–1.000)	0.228
hsCRP	0.993 (0.931–1.059)	0.820	0.939 (0.864–1.020)	0.138

CI—confidence interval, hsCRP—high-sensitivity C-reactive protein, IL—interleukin, NA—not applicable, NT-proBNP—N-terminal pro-brain natriuretic peptide, and OR—odds ratio.

## Data Availability

Anonymized data is available upon request from the corresponding author.
